# Myoclonus in Pediatric Metabolic Diseases: Clinical Spectrum, Mechanisms, and Treatable Causes—A Systematic Review

**DOI:** 10.3390/metabo16020098

**Published:** 2026-01-28

**Authors:** Elżbieta Majewska, Zofia Zdort, Aleksandra Ochocka, Justyna Paprocka

**Affiliations:** 1 Students’ Scientific Society, Pediatric Neurology Department, Faculty of Medical Sciences, Medical University of Silesia, 40-752 Katowice, Poland; s85978@365.sum.edu.pl (E.M.); s86359@365.sum.edu.pl (Z.Z.); s86048@365.sum.edu.pl (A.O.); 2 Pediatric Neurology Department, Faculty of Medical Sciences, Medical University of Silesia, 40-752 Katowice, Poland

**Keywords:** myoclonus, myoclonic seizures, inherited metabolic disorders, movement disorders, treatable neurometabolic disease

## Abstract

**Background**: Myoclonus, a sudden brief shock-like involuntary movement, represents a common yet under-recognized manifestation across many inherited metabolic disorders. Although its occurrence has been reported in case series and small cohorts, the overall spectrum, pathophysiological mechanisms, and therapeutic relevance of metabolic myoclonus have not been systematically summarized. **Methods**: A systematic search of PubMed was conducted for English-language publications from 2014 to 2025 using predefined MeSH terms related to myoclonus, movement disorders, and inborn errors of metabolism. Titles and abstracts were screened independently by three reviewers. After removal of duplicates, 27 articles were included, complemented by 65 additional references addressing individual disorders. Data were organized according to the International Classification of Inherited Metabolic Disorders (ICIMD). **Results**: Myoclonus was documented across six ICIMD categories, including intermediary metabolism, mitochondrial energy metabolism, lipid metabolism, disorders of complex molecules and organelles, cofactor and mineral metabolism, and metabolic cell signaling disorders. Clinical presentation ranged from isolated jerks to progressive myoclonic epilepsies. Several conditions—such as GLUT1 deficiency, cerebrotendinous xanthomatosis, and folate receptor α deficiency—are treatable through dietary or pharmacological interventions. **Conclusions**: Recognition of myoclonus as a presenting feature of inherited errors of metabolism (IEMs) is critical for timely diagnosis and treatment. Metabolic screening should be considered in all unexplained cases of myoclonus, particularly when accompanied by developmental delay or systemic abnormalities.

## 1. Introduction

Myoclonus is defined as a sudden, brief, shock-like involuntary movement caused by either bursts of muscle activity (positive myoclonus) or transient muscle inhibition (negative myoclonus). It is not a disease entity but a symptom that may occur at rest, during voluntary action, or in response to external stimuli such as auditory, tactile, or emotional triggers [1,2]. Myoclonus is typically irregular, lasting 10–50 ms, and may vary in distribution and amplitude. Depending on the anatomical generator, it can originate from cortical, subcortical or segmental (peripheral) structures. Although myoclonus is classically associated with epileptic and neurodegenerative conditions, it is also a prominent neurological sign in several inherited metabolic disorders [1,3]. The International Classification of Inherited Metabolic Disorders (ICIMD) lists over 1400 disorders grouped into 24 categories and more than 120 subgroups [4]. Many of these diseases can disrupt neuronal excitability and neurotransmitter balance, leading to myoclonic jerks, seizures, or progressive myoclonic epilepsies. Despite its clinical importance, the metabolic etiology of myoclonus remains underrecognized [2]. Reports are often limited to isolated case studies, and comprehensive analyses are lacking. Moreover, several metabolic diseases presenting with myoclonus, such as propionic acidemia, GLUT1 deficiency, or cerebrotendinous xanthomatosis, are treatable if diagnosed early, using dietary modification, vitamin or cofactor supplementation, or targeted pharmacotherapy [5]. This review aims to provide a systematic synthesis of the literature describing inborn errors of metabolism (IEMs) associated with myoclonus. We summarize the clinical, neurophysiological, and imaging characteristics of these disorders, discuss shared mechanisms of neuronal dysfunction, and highlight diagnostic and therapeutic strategies. Ultimately, our goal is to promote earlier recognition of metabolic causes in patients presenting with unexplained myoclonus and to emphasize treatable conditions within this broad group.

## 2. Materials and Methods

**Figure 1:**
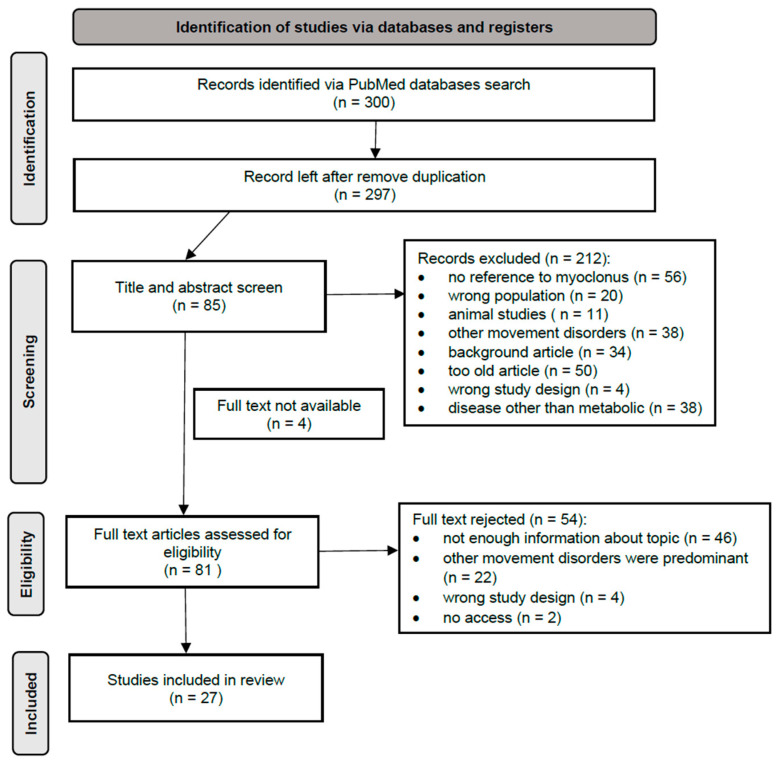
PRISMA flow diagram for screening and selection.

A comprehensive literature search was conducted using PubMed in March 2025. At the beginning of the project, we decided to use works from the last 10 years. However, several works from 2014 proved to be very helpful, so they were included. This search strategy included the following keywords along with their medical subject headings (MeSH terms): (“myoclonus” OR “myoclonic jerks” OR “involuntary movements” OR “movement disorders” OR “myoclonic seizures”) AND (“children” OR “pediatric” OR “infants”) AND (“metabolic disorders” OR “inborn errors of metabolism” OR “mitochondrial disorders” OR “lysosomal storage diseases” OR “urea cycle disorders” OR “organic acidemias” OR “aminoacidopathies” OR “peroxisomal disorders” OR “glycogen storage diseases” OR “fatty acid oxidation disorders” OR “purine metabolism disorders” OR “pyrimidine metabolism disorders” OR “congenital disorders of glycosylation” OR “sterol metabolism disorders” OR “neurotransmitter metabolism disorders” OR “Wilson disease” OR “Menkes disease” OR “homocystinuria” OR “galactosemia”). Only articles written in English and describing human subjects were included. Studies performed exclusively in animal models were excluded. Three independent reviewers screened all titles and abstracts. Following removal of duplicates, 297 records were screened, 85 underwent full-text evaluation, and 27 met inclusion criteria. This review was conducted in accordance with the PRISMA 2020 guidelines (Figure 1). An additional 65 references were used to complement disease-specific details, including biochemical and genetic information. Disorders were classified according to the International Classification of Inherited Metabolic Disorders (ICIMD). Data extracted from each publication included type and localization of myoclonus, EEG characteristics, MRI findings, biochemical diagnostics and treatment response. Given the heterogeneity of the study designs, a narrative synthesis rather than quantitative meta-analysis was performed.

## 3. Results

### 3.1. Intermediary Metabolism: Nutrient Pathways

This category encompasses disorders of amino acid, organic acid, and carbohydrate metabolism [4], where several disorders can include myoclonus as a symptom. A detailed list of diseases in this category and those described below can be found in Table 1.

Organic acidurias are characterized by abnormal excretion of organic acids in urine and include propionic acidemia and glutaric aciduria type I, both of which can manifest with myoclonic seizures [6,7,8].

Propionic acidemia results from mutations in PCCA or PCCB, leading to deficiency of propionyl-CoA carboxylase. Neurological manifestations result from accumulation of propionic acid with its metabolites and include developmental delay, hypotonia, encephalopathy, and multiple seizure types—particularly myoclonic, focal, and atypical absence seizures. EEG may demonstrate frontotemporal slow-wave activity and 7–9 Hz central comb-like rhythms. MRI often shows basal ganglia lesions, delayed myelination, or cerebellar atrophy [7,9]. Early dietary protein restriction and L-carnitine supplementation mitigate metabolic crises and improve neurological outcomes [10].

Glutaric aciduria type I arises from GCDH mutations leading to glutaryl-CoA dehydrogenase deficiency, causing accumulation of neurotoxic glutaric and 3-hydroxyglutaric acids [10]. Patients exhibit developmental delay, dystonia, and seizures, including myoclonic episodes. EEG often reveals temporoparietal discharges, while MRI demonstrates frontotemporal hypoplasia, subdural collections, and caudate-putamen signal abnormalities [7,8]. Early identification through newborn screening enables treatment with a low-lysine diet and carnitine supplementation, reducing the risk of encephalopathic crises and severe neurological damage [10].

GLUT1 deficiency syndrome, caused by SLC2A1 mutations, represents another treatable metabolic cause of myoclonus [11,12]. Impaired glucose transport across the blood–brain barrier leads to an energy-deficient brain state [6]. Clinically, patients present with infantile-onset seizures, developmental delay, microcephaly, and movement disorders including myoclonus and ataxia [6,11,12]. EEG may show generalized spike-and-wave discharges, and MRI can reveal white matter abnormalities or cerebellar atrophy [12]. The ketogenic diet remains the mainstay therapy, effectively controlling seizures and movement episodes [10].

In the non-classical GLUT1 phenotype, also known as paroxysmal exercise-induced dyskinesia (PED), patients experience transient myoclonic or dystonic movements provoked by exertion or fasting [5,10]. The modified Atkins diet and triheptanoin supplementation have shown efficacy in reducing episode frequency [10,11,13].

**Table 1:** Inherited metabolic disorders associated with myoclonus, summarized by ICIMD category.

Name of Disorder	*Mutation*	Type of Myoclonus	Age of Onset	Other Neurological Symptoms	Other Symptoms	EEG	MRI	Biomarkers	Treatment	Sources
INTERMEDIARY METABOLISM: NUTRIENT										
Propionic acidemia—1.2. organic acidurias	*PCCA*, *PCCB* (#606054)	myoclonic seizures	newborns (first days/weeks of life), late-onset	DD, dystonia, chorea, encephalopathic crisis, other types of seizures, optic atrophy, chronic neuropathy, hypotonia	vomiting, poor feeding, lethargy, dilated or hypertrophic cardiomyopathy, chronic gastrointestinal complaints	comb-like rhythm with 7–9 Hz central activity, background disorganization, frontotemporal and occipital slow-wave activity	basal ganglia changes, stroke-like episodes changes, delayed myelination, white matter changes, cerebral atrophy, cerebellar atrophy, cerebellar hemorrhage	screening: ↑ C3, urine organic acids, ↑ anion gap, hypoglycemia, hyperammonemia, hyperglycinemia, ↑ ALT, AST, neutropenia, thrombocytopenia, pancytopenia	avoid or treat triggers, dietary protein restriction, L-carnitine	[7,10,11]
Glutaric aciduria (acidemia) type I —1.2. organic acidurias	*GCDH* (#231670)	myoclonic seizures	infancy, early childhood (<2 years)	DD, dystonia, parkinsonism, choreoathetosis, encephalopathic crises, hypotonia, macrocephaly, dysarthria, subdural hemorrhages	retinal hemorrhages, early progressive, speech and feeding difficulties, metabolic crises	generalized slowing, focal temporoparietal epileptogenic discharge	enlarged perisylvian fissures, subdural effusion	↓ glutaryl-CoA dehydrogenase in skin	avoid or treat triggers, dietary lysine restriction, L-carnitine	[5,7,8,10,11]
Maple syrup urine disease (MSUD) —1.3. disorders of branched-chain amino acid metabolism	*BCKDHA* (type IA: #248600)	myoclonic seizures	newborns (first days of life)	neonatal encephalopathy, opisthotonus, stereotyped movements of limb (fencing, bicycling), ataxia, dystonia, tremor, DD, other types of seizures	irritability, poor feeding, lethargy, apnea, coma, ketonuria, maple syrup odor	comb-like rhythm with 7–9 Hz central activity, burst-suppression, hypsarrhythmia, diffuse slowing, loss of reactivity to auditory stimuli	diffused edema involving the white matter of cerebellum, brain stem, globus pallidus, internal capsule and thalamus which usually occurs in myelinated areas	↑ plasma alloisoleucine and BCAA with disturbing the 1:2:3 normal ratio of isoleucine:leucine:valine, BCKA in urine	low protein and leucine restricted diet, avoid or treat triggers, liver transplantation	[7,10]
	*BCKDHB* (type IB: #620698)									
	*DBT* (type II: #620699)									
Phenylketonuria —1.4. disorders of phenylalanine and tyrosine metabolism	*PAH* (#261600)	myoclonic seizures	newborns, infants	DD, increased tendon reflex, ankle and patellar clonus, spasticity, parkinsonism, tremor, hypertonia, paraplegia/hemiplegia, progressive supranuclear motor disturbance, choreiform or athetoid hyperkinesia, optic atrophy, microcephaly	musty odor, craniosynostosis, features of Antley-Bixler syndrome	burst-suppression, hypsarrhythmia, epileptic spasms, diffuse background slowing, focal sharp waves, irregular generalized spikes and slow waves	delayed myelination, reduced brain size, astrocytic gliosis especially in optic tract, corpus callosum, subcortical white matter, periventricular white matter, cortico-hippocampal relay circuits and axonal connections to the prefrontal cortex	↑ Phe in blood, ↑ Phe/Tyr ratio	low Phe diet, sapropterin, sepiapterin, pegvaliase	[7,10]
Glycine encephalopathy 1/nonketotic hyperglycinemia —1.6. disorders of glycine and serine metabolism	*GLDC* (#605899)	myoclonic seizures, myoclonic jerks	newborns, infants (<3 months)	acute epileptic encephalopathy, seizures, infantile spasms, DD, hypotonia, mild intellectual disability, microcephaly	weakness, tone changes, respiratory compromise, vomiting, hypotonia, apneas, vertical gaze palsies, progressive lethargy, coma, poor feeding, hiccups	hypsarrhythmia, burst-suppression, multifocal epileptiform activity	non-specific changes or agenesis of corpus callosum, brain atrophy with ventriculomegaly, bilateral subcortical heterotropia, delayed myelination	↑ Gly in serum and CSF, ↑ CSF/plasma Gly ratio	sodium benzoate, dextromethorphan	[7,8,14,15]
Serine Biosynthesis Defects—1.6. disorders of glycine and serine metabolism	*PHGDH* (#601815)	myoclonic seizures	newborns, infants (first months of life)	DD, hypertonia, ataxia, polyneuropathy, epilepsy, other types of seizures, microcephaly	IUGR, congenital cataracts, feeding difficulties, hypogonadism	hypsarrhythmia, burst-suppression, multifocal spikes and sharp waves	hypomyelination, brain atrophy, hypoplastic cerebellum and pons	↓ Ser and Gly in plasma and CSF	Ser and Gly supplementation	[7]
GLUT-1 deficiency —3.6. disorders of carbohydrate transmembrane transport and absorption	*SLC2A1* (type I #606777)	myoclonus, myoclonic seizure, JME	early infancy (<4 months)	infantile-onset epilepsy/seizures, spasticity, ataxia, dystonia, chorea, tremor, DD, hypotonia, migraine/recurrent headaches, paroxysmal eye-head movement, alternating hemiplegia	abdominal pain, dyschromatopsia with retinal apigmentation, particular behavioral traits with friendly disposition, excessive communicative and jovial behavior, impulsivity or hyperactivity	non-specific changes or focal, generalized slowing or attenuation, generalized, focal or multifocal 2.5–4 Hz spike-and-wave discharges	non-specific changes or focal, diffuse hypersignal of supra-tentorial white matter on the T2/FLAIR sequence, an enlargement of Virchow-Robin spaces, ventricular dilatation, dysmorphic corpus callosum	CSF/plasma glucose ratio < 0.4 (hypoglycorrachia)	ketogenic (or modified Atkins) diet	[5,7,10,11,12]
PED = paroxysmal exercise-induced dyskinesia	(type II #612126)	myoclonus, myoclonic seizures	childhood, early adolescence	strabismus, pyramidal signs, brisk reflexes, dysarthry, swallowing difficulty, microcephaly, language delay, dysarthria, sleep disturbances				slightly ↓ CSF-to-blood glucose ratio	ketogenic diet, triheptanoin	
Carnitine acylcarnitine translocase deficiency —4.1. disorders of carnitine metabolism	*SLC25A20* (#212138)	myoclonic seizures	newborns, early infancy (first months of life)	acute encephalopathy, hypotonia, DD, lethargy	rhabdomyolysis, cardiac arrhythmia, poor feeding, transaminitis, liver dysfunction with hepatomegaly, hypertrophic cardiomyopathy, respiratory distress	generalized slowing, focal temporoparietal epileptogenic discharge	cerebral edema, intracranial bleeding, acute ischemia, moderate cortical loss with delayed myelination	hyperammonemia, hypoketotic, hypoglycemia,↑ long-chain acylcarnitines C16, C18 and C19:1, metabolic acidosis, ↑ lactate, ↑ ALT, AST	high carbohydrate diet, restriction of long-chain dietary fat, triheptanoin	[7,8]
Early Onset Multiple Carboxylase Deficiency —4.2. disorders of mitochondrial fatty acid oxidation	*HLCS* (#253270)	multifocal myoclonic seizures	newborns, early infancy	hypotonia, seizures, impaired consciousness	tachypnea, lethargy, vomiting, erythrodermic dermatitis, alopecia, immunosuppression	burst-suppression, multifocal epileptiform activity	antenatally: subependymal cysts, ventriculomegaly, intraventricular hemorrhage, IUGR	ketoacidosis, hypoglycemia	biotin supplementation	[7]
INTERMEDIARY METABOLISM: ENERGY										
Pyruvate dehydrogenase deficiency— 5.1. disorders of pyruvate metabolism	*PDHA1* (*300502)	myoclonic seizures	median age ~20 months	DD, epileptic spasms, chronic or paroxysmal dystonia, ataxia	encephalopathy, metabolic crises	multifocal slow spike-wave discharges, hypsarrythmia	accentuation of cortical sulci, hyperintensity in the bilateral basal ganglia region and lenticular nucleus	↑ lactate and pyruvate in blood, ↓ lactate:pyruvate ratio in CSF	ketogenic diet, triheptanoin treatment, thiamine supplements	[7,8,16,17,18,19]
Cerebral creatine deficiency type 2 —5.3. disorders of creatine metabolism	*GAMT* (#612736)	myoclonic seizures	early infancy to age 2 years	chorea, dystonia, truncal ataxia, head nodding, tremor, drop attacks, DD, myopathy	behavioral deficits	generalized paroxysmal spike-wave and slow-wave discharges with slowing in background activity in addition to generalized delta activity	non-specific changes	absence of creatine peak on proton MRS, low serum creatinine (or altered creatine concentrations in plasma)	creatine ± ornitine ± sodium benzoate, dietary restriction of arginine	[10,11,20,21]
MERRF— myoclonic epilepsy with ragged-red fibres—4.3.1. mitochondrial respiratory chain disorders	many genes e.g., *MTTK*, *MTTL1*, *MTTH*, *MTTS1*, *MTTS2*, *MTTF*, *MTDN5* (#545000)	myoclonus, myoclonic seizures, PMEs	from childhood to adulthood (usually before the age of 20)	hearing loss, peripheral neuropathy, cognitive decay, dementia, optic atrophy, encephalopathy, ataxia, cardiomyopathy, pigmentary retinopathy, pyramidal signs, ophthalmoparesis	short stature, exercise intolerance, weakness, tone changes, vomiting, respiratory compromise, al signs, ophthalmoparesis, appearance of multiple lipomas (neck and upper trunk)	generalized spike-and-wave discharges with background slowing, focal epileptiform discharges	brain atrophy and basal ganglia calcifications	↑ blood level of pyruvate and lactate, ↑ CSF protein level, myopathic pattern in EMG, ragged-red fibres in muscle biopsy	symptomatic	[7,22,23,24,25]
POLG-related disorders —4.3.1. mitochondrial respiratory chain disorders:	*POLG* (*174763)								symptomatic	[7,26,27,28,29,30]
AHS = Alpers- Huttenlocher syndrome		myoclonus, myoclonic seizures	prior to age 12 years	stroke, stroke-like episodes, neurodevelopmental regression, headaches, choreoathetosis, neuropathy, ataxia, areflexia, hypotonia, loss of cognitive function, vision loss, hearing loss	liver failure, exercise intolerance	high-amplitude slow activity with smaller polyspikes or intermittent continuous spike-wave activity/may be normal or show only focal slowing of the background rhythm	may be normal/ atrophy of both occipital lobes, high signal intensity in occipital lobe white matter	↑ GGTP, ALT, AST		
MEMSA/ SCAE = myoclonic epilepsy myopathy sensory ataxia/spino- cerebellar ataxia with epilepsy		myoclonic seizures	12–40 years	myopathy, epilepsy, and ataxia without ophthalmoplegia, progressive interictal encephalopathy, migraine headaches		intermittent runs of rhythmic delta activity	bilateral occipital lesions around calcarine sulci	↑ lactate, Ala		
ANS = ataxia neuropathy spectrum		myoclonus	12–40 years	occipital stroke, visual field deficit, peripheral neuropathy, migraine, epilepsy, ataxia	valproate-induced hepatic necrosis	diffuse delta wave slowing, diffuse cerebral dysfunction	hyperintensity signal and volume loss in left > right occipital lobes consistent with prior infarcts	↑ CSF protein, serum lactate and ALT, AST		
LIPID METABOLISM AND TRANSPORT										
Cerebrotendinous Xanthomatosis —14.8. disorders of bile acid metabolism	*CYP27A* (#213700)	myoclonus	neurological symptoms in adulthood, other symptoms manifesting in infancy/ childhood	corticospinal tract dysfunction with spasticity, hyper-reflexia, dystonia, parkinsonism, progressive ataxia with spasticity, pyramidal signs, neuropathy, cognitive decline, other types of seizures, peripheral neuropathy	xanthomas near large tendons, cognitive decline, psychiatric symptoms, neonatal cholestatic jaundice, bilateral childhood- onset cataracts, chronic diarrhea	derangements with irregular slow theta and delta waves and frequent bursts of high-voltage activity	cortical and cerebellar atrophy, white matter signal alterations and symmetric hyperintensities in the dentate nuclei	↑ plasma cholestanol levels, ↑ bile alcohols in plasma and urine	chenodeoxycholic acid	[5,10]
COMPLEX MOLECULE AND ORGANELLE METABOLISM										
Congenital defects of glycosylation—18. congenital disorders of glycosylation:								screening: transferrin isoforms analysis		
PMM2- CDG/ CDG-Ia	*PMM2* (*601785)	myoclonus, myoclonic seizures	infants, childhood (3–10 years)	ESE, other types of seizures, DD, DD/ID, cerebellar ataxia, peripheral neuropathy, hyperkinetic movement disorders, stroke-like episodes, retinis pigmentosa, hypotonia, hyporeflexia	faltering growth, abnormal subcutaneous fat distribution, characteristic facial features, hypoglycemia, hypothyroidism, osteopenia, pericardial effusions, risk of lifelong bleeding, recurrent upper respiratory tract infections	hypsarrythmia, focal slowing with diffuse slowing, focal epileptiform activity, generalized epileptiform activity	cerebellar hypoplasia/ atrophy, small brain stem	↑ ALT, AST, coagulopathy with abnormal prothrombin time, ↓ serum concentration of factors IX and XI, antithrombin III, protein C, and/or protein S, proteinuria, aminoaciduria	symptomatic	[7,8,10,31,32,33,34,35,36,37,38,39,40,41,42,43]
ALG11- CDG/CDG-Ip	*ALG-11* (*613666)	myoclonic seizures	infants (<6 months)	DD, epilepsy, hypotonia, hypertonia, microcephaly	dysmorphic features, feeding problems, eye/visual problems, deafness	hypsarrythmia suppression burst activity, modified hypsarrhythmia with repeated bilateral spikes in temporal and central regions	reduced diffusion along the periventricular parietal and temporal white matter tracts as well as the splenium of the corpus callosum	non-specific	symptomatic	
ALG-13- CDG	*ALG-13* (*300776)	myoclonic-tonic spasms	infants (<6 months)	DD, DD/ID epilepsy, hypotonia	coagulation abnormalities, endocrine dysfunction	hypsarrhythmia, multifocal discharges	non-specific changes or cerebral atrophy, benign enlargement of the subarachnoid spaces	non-specific	symptomatic	
PIGA- CDG	*PIGA* (#300868)	myoclonic seizures	newborns, early infancy	other types of seizures, gelastic epilepsy, DD, DD/ID, global developmental delays, hypotonia, hypertonia, dystonia, cortical visual impairment, sleep disorders	congenital heart disease, feeding difficulties, respiratory complications	hypsarrhythmia, diffuse background slowing, focal slowing, and multifocal or diffuse spike/sharp and slow waves	delayed myelination, cerebellar hypoplasia, abnormal corpus callosum, small optic nerves, cortical dysplasia, restricted diffusion, prominent cortical and subcortical volume loss with brainstem atrophy	non-specific	symptomatic	
PIGN- CDG	*PIGN* (*606097)		newborns	other types of seizures, DD, hypotonia	facial features, underdeveloped fingertips, GI reflux	diffuse slow waves with multifocal discharges dominated	cerebellar atrophy, small corpus callosum, cerebral volume loss, abnormal/delayed myelination	non-specific	symptomatic	
NGLY1 deficiency	*NGLY1* (*610661)	myoclonic seizures	newborns	other types of seizures, epilepsy, DD, hypotonia, movement disorder, microcephaly, tremor, ataxia, ocular apraxia	dysmorphism, constipation, lacrimal hyposecretion, corneal ulceration	diffuse multifocal spike-and-wave complex formation, high-amplitude SEP	cerebral and cerebellar atrophy	↑ ALT, AST	perampanel	[44,45,46]
Zellweger syndrome	different mutations in *PEX gene* e.g.,	myoclonic seizures	newborns, late-infantile, early-childhood (sometimes adulthood)	DD, hypotonia, ataxia, epilepsy, sensorineural deafness	prolonged jaundice, dysmorphic features, retinopathy, cataracts, glaucoma, early blindness, tunnel vision, hepatic dysfunction, feeding difficulties, coagulopathy, renal calcium oxalate stones, adrenal insufficiency	hypsarrhythmia, burst-suppression, multifocal epileptiform activity	neocortical dysplasia, generalized decrease in white matter volume, delayed myelination, bilaterial ventricular dilatation, germinolytic cysts, leucodystrophy	↑ plasma— very-long-chain fatty acids, D and trihydroxy-cholestanoic, phytanic, pristanic and pipecolic acids, ALT, AST, bilirubin, erythrocytes—↓ plasmalogens, ↑ urine—di- and trihydroxy-cholestanoic and pipecolic acids, abnormal skin fibroblast analysis	symptomatic	[7,8,47,48]
	*PEX1* (*6021361)									
	*PEX13* (*601789)									
	*PEX19* (*600279)									
	*PEX26* (*608666)									
Gangliosidosis —20.1. disorders of sphingolipid degradation										
GM1	*GLB1* (#230500)	myoclonic seizures	type I—infants (<6 months)	developmental regression, ataxia, impairment of gross and fine motor skills, dysarthria	macular cherry-red spots, hepato- splenomegaly, hypertrophic/dilated, cardiomyopathy, coarse facial features, generalized skeletal dysplasia, corneal clouding	diffuse irregular slow activity	progressive and diffuse atrophy	↓ beta- galactosidase, melanocytosis (Mongolian spots), ↑ AST (normal ALT), ↑ chitotriosidase activity, vacuolated lymphocyte, abnormally granulated eosinophils in peripheral blood smear	symptomatic	[7,8,19,49,50,51,52]
			type II—late-infantile (1–3 years), childhood (3–10 years)							
			type III—adulthood (<30 years)							
GM2/ Sandhoff disease	*HEXB* (#268800)	myoclonic seizures	type I—infants (3–6 months)	progressive weakness or loss of motor skills, decreased attentiveness, exaggerated startle response, hypotonia, hyperreflexia, seizures, developmental plateauing followed by regression, progressive spasticity, dysarthria, dysphagia	cherry-red macula, progressive macrocephaly, hepato-splenomegaly (late-onset—absence of hepato-splenomegaly)	generalized slowing, focal temporoparietal epileptogenic discharge	hyperintense signal in the external capsule and cerebellar white matter, brain atrophy	↓ HEX A and HEX B activity (acute infantile type, some residual HEX A and HEX B activity in subacute or late-onset type)		
			type II— childhood (2–10 years)							
			type II—adulthood (20–30 years)							
Gaucher disease type III—20.1. disorders of sphingolipid degradation	*GBA* (#231000)	myoclonic seizures	childhood (2–15 years)	progressive myoclonic epilepsy, ataxia, spasticity, oculomotor apraxia	weakness, tone changes, vomiting, respiratory compromise, encephalopathy, hepatomegaly, splenomegaly, pain crises, pathologic fractures	diffuse polyspikes discharges with occipital predominance, rhythmic runs of 6–10 Hz spikes or sharp waves	mild cerebral atrophy	cytopenia, abnormal wave forms in BAER	miglustat	[7,10,53]
Sialidosis type I—20.3. disorders of glycoprotein degradation	*NEU1* (*608272)	myoclonic seizures, PMEs	childhood, adulthood (12–25 years)	ataxia, seizures and jerks, nystagmus	cherry red spot on the macula, impaired vision, muscle pain, gait disturbance, hearing loss, dysarthria	cortical myoclonus with epileptic or discharges that are time-locked with muscle bursts	moderate diffuse brain atrophy	non-specific	symptomatic	[54]
Neuronal ceroid lipofuscinosis—20.4. neuronal ceroid lipofuscinosis										
CLN2	*TPP1* (#204500)	myoclonic seizures	4–8 years	developmental regression, ataxia, gross motor functions loss, vision loss, dystonia, microcephaly	feeding and swallowing difficulties	photoparoxysmal response to low frequency (1–2 Hz) intermittent photic stimulation, time-locked response to 1 Hz photic stimulation consisting of bioccipital, generalized spike-and-wave discharges	cerebellar and vermian atrophy	↓ tripeptidyl peptidase 1 activity (DBS, saliva, leucocytes, fibroblasts)	cerliponase alfa	[7,8,27,55,56,57,58,59,60,61,62,63]
CLN6A	*CLN6* (#601780)	myoclonic seizures	from 18 months to 8 years	developmental regression, vision loss, ataxia, microcephaly, pyramidal signs, sleep disturbances		epileptiform discharges, low-frequency intermittent photic stimulation	diffuse cerebral and cerebellar atrophy	non-specific	non-specific	
CLN11	*CLN11* (#614706)	myoclonic seizures	from 5 to 25 years	retinal dystrophy, cataracts, cognitive decline, visual hallucinations, pyramidal signs		epileptiform discharges	cerebellar atrophy	non-specific	non-specific	
CLN14	*KCTD-7* (*611725)	PMEs	14 months	other types of seizures, neurological regression, cognitive regression, dysarthria, ataxia, hypotonia, motor impairment, loss of speech		non-specific	cortical and cerebellar atrophy, white matter signal alterations and symmetric hyperintensities in the dentate nuclei	non-specific	non-specific	
Niemann-Pick disease type C—20.6. other disorders of complex molecule degradation	*NPC1* (#257220)	myoclonus	early-infantile (<2 years), late-infantile (<6 years), juvenile (<15 years), adolescent (>15 years)	ataxia, spasticity, dystonia, vertical supranuclear gaze palsy, intellectual disability, dysphagia, dysarthria	hepatosplenomegaly, hepatic dysfunction, gelastic cataplexy, acute psychosis, depression, obsessive- compulsive disorder, socially inadequate or dysinhabited behaviors	diffuse background slowing and interictal discharges, normal in gelastic cataplexy and shows high-frequency oscillations	diffuse cerebral atrophy	↑ oxysterols, lyso-sphingomyelin derivatives and bile acids, ↓ leukocyte sphingomyelinase	miglustat, aqneursa	[5,8,10,55,64,65,66]
	*NPC2* (5%) (#607625)									
COFACTOR AND MINERAL METABOLISM										
Tetrahydrobiopterin deficiency—21.1. disorders of tetrahydrobiopterin metabolism	*GCH1* (#233910)	(less frequent) myoclonic seizures—more frequent in dihydropteridine reductase and 6-pyruvoyl-tetrahydropterin synthase deficiency, myoclonic jerks	infancy	floppy baby, hypotonia of the trunk, hypertonia of the extremities, swallowing	behavioral problems, poor sucking	non-specific changes	patients with HPA—hyperintensity in T2-fluid attenuated inversion recovery sequences in the bilateral cerebral white	↑ Phe levels in NBS or selective diagnostic work-up in patients with AR-GTPCHD, PTPSD, DHPRD or PCDD, abnormal levels pterins in urine and DBS	Phe-reduced diet, sapropterin dihydrochloride, L-Dopa with peripheral DC inhibitor (carbidopa or benserazide), 5-HTP, folinic DA, MAO-, anticholinergic agents, COMT inhibitors, SSRIs, benzodiazepines, melatonin, botulinum toxin injections	[67]
Cerebral Folate Transport Deficiency—21.8. disorders of folate metabolism	*FOLR1* (# 613068)	myoclonic seizures, myoclonic jerks	>1 year	ataxia, dyskinesia, dystonia, spasticity, seizures, DD/ID	psychiatric impairment	multifocal epileptiform activity, diffuse background slowing	profound hypomyelination and atrophic changes in cerebral and cerebellar cortex depletion of white matter	↓ 5-MTHF in CSF	folinic acid	[5,7,10]
	*SLC46A1* (#229050)									
X-Linked Cobalamin Disorder—21.9. disorders of cobalamin metabolism	*HCFC1* (#309541)	myoclonus seizures	prenatal period to 5 months	epileptic encephalopathy with early tonic, clonic seizures, brisk knee jerk reflexes, Babiński signs, severe neurocognitive delay	hypotonia, poor visual pursuit, apathy	bilateral paroxysmal activity during seizures	high signal intensity with both restricted diffusion and decreased apparent diffusion in the posterior limb of internal capsule that later progressed over the entire internal capsule and posterior white matter	↑ plasma and urine methylmalonate, potentially low plasma methionine	non-specific	[68]
Pyridoxine metabolism—21.6. disorders of pyridoxine metabolism:										
Pyridoxine -dependent epilepsy	*ALDH7A1* (#266100)	myoclonic jerks	first hours of life	encephalopathy, other types of seizures, unusual eye movements or facial grimacing, neurodevelopmental disability, autistic features, and other behavioral problems, DD, affecting speech, cognition, and behavior, hypotonia, dystonia	congenital cataracts and facial dysmorphism that includes hypertelorism, depressed nasal bridge, epicanthal folds, high hairline, pointed chin, full eyebrows, and broad nasal root respiratory distress, anemia, failure to gain weight, abdominal distention, poor feeding	burst-suppression, discontinuous background with multifocal spikes, and later hypsarrhythmia in some infants	frequently abnormal, agenesis or hypoplasia of the corpus callosum, ventriculomegaly, non-specific white matter signal change, mega cisterna magna and cerebellar hypoplasia	↑ α-AASA, pipecolic acid in urine or blood	pyridoxine supplementation, lysine restriction, and L-arginine supplementation	[7,14]
Pyridoxal phosphate- responsive epilepsy	*PNPO* (#603287)		classic PNPO deficiency— <20 weeks, sometimes even manifesting in utero			burst-suppression and hypsarrhythmia, multifocal epileptiform discharges and generalized spike-wave can also occur	cerebral edema, basal ganglia or white matter signal abnormalities, delayed myelination or hypomyelination, intraventricular hemorrhage, arterial infarction and simplified gyral patterns		pyridoxal 5′-phosphate	
			late-onset— after neonatal period (only 7 cases reported)							
Methylenetetrahydrofolate Reductase Deficiency—21.8. disorders of folate metabolism	*MTHFR* (#236250)	myoclonic seizures	neonatal period, cases of adult onset (around 20 years old) have been reported	neonatal encephalopathy with hypotonia, feeding difficulty, microcephaly, other types of seizures, DD	hematological abnormalities reported in older patients	diffuse background slowing, continuous spike-wave complexes or multifocal spikes	white matter predominant leukoencephalopathy, periventricular or subcortical, sometimes with sparing of U-fibres, delayed or absent myelination and cerebral atrophy	↑ homocysteine with low or low-normal methionin, no methylmalonic acid elevation	betaine, hydroxocobalamin, folate (particular forms)	[7]
Biotinidase Deficiency—21.7. disorders of biotin metabolism	*BTD* (#253260)	myoclonic seizures	1 week–10 years, mean age of onset 3.5 months	ataxia, spastic paresis, dystonia, seizures, DD	visual and auditory impairment, skin changes, alopecia, hypopigmented skin	burst-suppression, poorly organized and slow awake background, lack of typical sleep elements, frequent spikes and spike-slow-wave discharges, generalized slowing	age-dependent symmetric white matter abnormalities with T2 hyperintensity, delayed myelination, and cerebral and cerebellar atrophy or swelling, brainstem involvement including periaqueductal gray matter, dorsal pons, medulla, and cerebellar peduncles, restricted diffusion, basal ganglia involvement, optic pathway abnormalities, and possible spinal cord involvement	↓ serum biotinidase	biotin	[7,8,10,49,69]
Menkes Disease—22.1 disorders of copper metabolism	*ATP7A* (#309400)	stimulation-induced myoclonic jerks, myoclonic seizures	<3 years	hypotonia, loss of milestones, refractory seizures and failure to thrive, followed by progressive neurodegeneration, subdural hemorrhages, macrocephaly, hypotonia	retinal hemorrhages, macrocephaly, hypopigmented brittle hair, fractures, hypotonia	hypsarrythmia, multifocal spike and slow-wave activity, burst-supression, generalized slowing	subdural effusion	↓ serum copper and ceruloplasmin—unreliable in early diagnosis, abnormal plasma catechol concentrations in males with classic Menkes disease	non-specific	[7,8]
METABOLIC CELL SIGNALING										
Succinic Semialdehyde dehydrogenase deficiency (SSADH)—23.2 gamma-aminobutyric acid neurotransmitter disorders	*ALDH5A1* (#271980)	myoclonic seizures	<2 years	focal motor, absence seizures, non-progressive encephalopathy, hypotonia, developmental delay, autism, ataxia, dystonia, dyskinesia, hyporeflexia, sleep disturbances	attention problems, anxiety, obsessive-compulsive behaviors	diffuse background slowing, focal and multifocal interictal epileptiform activity	T2-weighted signal involving globus pallidus, cerebellar dentate nucleus and subthalamic nucleus, cerebral and cerebellar atrophy, delayed myelination	↑ GHB on urine, absence of metabolic acidosis	symptomatic	[7]
Dopa-responsive dystonia (DRD)—23.1. monoamine neurotransmission	*TH* (#605407)	myoclonus	<5 years	hypokinesia, bradykinesia, tremor, dystonia, parkinsonism, delayed motor development, spasticity, hypotonia, encephalopathy, oculogyric crises type A: lower limb dystonia, rigidity type B: focal or generalized dystonia, intellectual impairment postural dystonia of extremities, brisk deep tendon reflexes, pyramidal signs atypical: action dystonia— retrocollis, oculogyric crises, postural tremor, parkinsonism	autonomic dysfunction, ptosis	normal	non-specific changes or bilateral widening of the frontotemporal extracerebral space, ventriculomegaly, other non-specific changes	hyperprolactinemia, ↓ HVA in CSF normal phenylalanine levels in blood (comparing to other DRD)	L-dopa type A: low doses—good response type B: worse response, more sensitive to L-dopa	[1,5]
	*GCH1* (#128230)				inward rotation of the feet, postural instability, depression, anxiety		non-specific changes	↓ CSF level of HA, biopterin, neopterin with normal plasma level of Phe	L-dopa/ carbidopa	

### 3.2. Energy Metabolism: Mitochondrial Disorders

Mitochondrial diseases affect oxidative phosphorylation and energy production [4]. Within this group, myoclonic epilepsy with ragged-red fibres (MERRF) and POLG-related disorders are the principal syndromes associated with myoclonus.

MERRF is caused by pathogenic variants in mitochondrial tRNA genes, most commonly m.8344A > G in MT-TK [22]. The classical phenotype includes myoclonus, generalized epilepsy, ataxia, and ragged-red fibres on muscle biopsy. Myoclonic seizures are the hallmark, often accompanied by generalized spike-and-wave EEG patterns with background slowing. MRI typically demonstrates brain atrophy and basal ganglia calcifications [23]. Laboratory findings include elevated lactate and CSF protein levels. Treatment is supportive, focusing on seizure control, and valproic acid should be avoided, especially in coexistent POLG mutations. Some benefit has been observed with coenzyme Q10, idebenone, and L-carnitine supplementation, although evidence remains limited [22]. While MERRF is the paradigmatic “myoclonic” mitochondrial disease, recent evidence underscores the need to differentiate it from MELAS. According to GeneReviews, myoclonus occurs in 25–49% of patients with MELAS, making it a frequent but not defining feature, unlike in MERRF where it is a core symptoms [70]. In MELAS, focal seizures and stroke-like episodes predominate, whereas MERRF is characterized by generalized myoclonus and ataxia. Understanding these phenotypic overlaps and distinctions is crucial, as both conditions require avoidance of mitochondrial toxins (e.g., valproic acid) and may benefit from metabolic stabilization.

POLG-related disorders arise from mutations in the POLG gene encoding mitochondrial DNA polymerase gamma. These include a broad clinical spectrum: AHS (Alpers-Huttenlocher syndrome), presenting in early childhood with intractable myoclonic epilepsy, liver failure, and encephalopathy; MEMSA (myoclonic epilepsy, myopathy, sensory ataxia), with generalized myoclonus and progressive ataxia; and ANS (ataxia neuropathy spectrum), characterized by cerebellar ataxia, neuropathy, and occasional myoclonus [26]. EEG findings and MRI changes vary according to the phenotype and are described in detail in Table 1. Prognosis depends on age of onset and disease severity. There is no curative treatment; symptomatic management and avoidance of valproate are essential to prevent hepatic failure [26].

### 3.3. Lipid Metabolism and Transport

Disorders of lipid metabolism and transport comprise abnormalities in fatty acid oxidation, bile acid synthesis, and sterol metabolism [4]. Among these, cerebrotendinous xanthomatosis (CTX) stands out as a prominent cause of myoclonus within this group.

CTX results from CYP27A1 mutations leading to impaired bile acid synthesis and accumulation of cholestanol and cholesterol [71]. The disease presents with a wide clinical spectrum that includes childhood-onset cataracts, chronic diarrhea, tendon xanthomas, and progressive neurological decline in adolescence or adulthood. Neurological involvement is marked by ataxia, parkinsonism, cognitive impairment, and myoclonus, which may mimic progressive myoclonic epilepsy [71,72].

Electrophysiological recordings confirm subcortical myoclonus originating from dentate-basal ganglia dysfunction. MRI findings often reveal cerebellar and cortical atrophy and symmetric dentate nucleus hyperintensities [73]. Importantly, CTX is treatable: administration of chenodeoxycholic acid (CDCA) normalizes bile acid metabolism, reduces cholestanol accumulation, and can stabilize or even reverse neurological symptoms, including myoclonus. Early treatment is associated with the most favorable outcomes [10].

### 3.4. Complex Molecule and Organelle Metabolism

This category consists of congenital disorders of glycosylation (CDG), peroxisomal and lysosomal diseases, and defects in organelle biogenesis. Myoclonus or myoclonic seizures have been described in several entities: PMM2-CDG, ALG11-CDG, ALG-13-CDG, PIGA-CDG, PIGN-CDG, NGLY1 deficiency, Zellweger spectrum disorders, gangliosidosis type 1 and 2, Gaucher disease type III, sialidosis type I, ceroid lipofuscinosis 2, 6A, 11, 14 and Niemann-Pick disease type C (NPC).

NGLY1 deficiency together with sialidosis, neuronal ceroid lipofuscinosis and MERRF syndrome forms a group of heterogeneous disorders called Progressive Myoclonus Epilepsies (PME). They are characterized by myoclonus, generalized epilepsy and neurological deterioration, including dementia and ataxia [74]. NGLY1 deficiency in particular is caused by mutations in NGLY1, which encodes N-glycanase 1—an enzyme responsible for deglycosylating misfolded glycoproteins prior to proteasomal degradation [45]. Disruption of this pathway leads to accumulation of aberrant glycoproteins and impaired cellular stress responses. Clinically, affected patients exhibit hypo- or alacrimia, hypotonia, peripheral neuropathy, microcephaly, liver dysfunction (indicated by elevated serum transaminases and liver fibrosis) and myoclonic seizures. EEG findings show multifocal epileptiform discharges, particularly in frontocentral regions, often correlated with jerks on video–EEG [46]. MRI frequently reveals progressive cerebral and cerebellar atrophy and delayed myelination. Treatment is symptomatic, though emerging studies suggest potential benefit from AMPA receptor antagonists such as perampanel, which may improve action myoclonus [75].

Zellweger syndrome represents the severe end of the spectrum of peroxisomal biogenesis disorders (PBDs), standing in contrast to single-enzyme peroxisomal defects [47,48]. It is caused by mutations in PEX genes essential for organelle assembly, leading to defective formation and function of peroxisomes. While myoclonus has been linked to PEX13, PEX19, and PEX26, it is also observed in phenotypes associated with other genes, including PEX1. The clinical presentation includes global developmental delay, hypotonia, ataxia, epilepsy, myoclonic seizures, sensorineural hearing loss, and ocular abnormalities such as retinopathy, cataracts, glaucoma, progressive blindness and tunnel vision. EEG often reveals burst-suppression or hypsarrhythmic patterns, consistent with severe epileptic encephalopathy. MRI shows white matter hypomyelination, neocortical dysplasia, delayed myelination, germinolytic cysts, leukodystrophy and ventriculomegaly. Biochemically, patients exhibit elevated very-long-chain fatty acids and abnormal bile acid intermediates. Deficiency of plasmalogens is detectable in erythrocytes, and diagnostic confirmation is obtained through biochemical testing and skin fibroblast analysis [71]. There is currently no curative therapy; management is supportive, focusing on seizure control and nutritional support.

Niemann-Pick disease type C (NPC) is a lysosomal storage disorder caused by mutations in NPC1 (95%) or NPC2 leading to defective intracellular lipid trafficking and accumulation of cholesterol and glycosphingolipids [48]. It may manifest itself as progressive cognitive decline (78%), gait and limb ataxia (70%), dysarthria, dystonia, dysphagia, and cataplexy. Vertical supranuclear gaze palsy is a robust clinical indicator of NP-C [64]. EEG often reveals generalized slowing and interictal discharges, while MRI demonstrates diffuse cerebral atrophy [64]. Biochemical markers such as oxysterol, bile acid derivatives and lyso-sphingomyelin-509 aid diagnosis. The only currently approved treatment with miglustat, an inhibitor of glycosphingolipid synthesis, slows neurological progression. Gene therapy and combination strategies are under investigation, but early initiation of treatment appears critical to improving outcomes [65]. While miglustat, an inhibitor of glycosphingolipid synthesis, is approved in many jurisdictions (including Europe and Japan) to slow neurological progression, it is not FDA-approved for NPC in the United States. Recently, regulatory agencies have authorized new agents; the United States FDA has approved both levacetylleucine and arimoclomol for the treatment of NPC. In Europe, the EMA recommended granting marketing authorization for levacetylleucine (Aqneursa) in July 2025. It is indicated for the treatment of neurological manifestations and, in the EU, is used in combination with miglustat, or as a monotherapy in patients where miglustat is not tolerated. Preclinical studies suggest that levacetylleucine enhances ATP production and mitochondrial energy metabolism, contributing to improved motor function and reduced myoclonus.

### 3.5. Cofactor and Mineral Metabolism

Cofactor and mineral metabolism disorders are inherited neurometabolic conditions broadly divided into two groups: disorders of vitamin and cofactor metabolism, and disorders of trace element and metal metabolism. In our review, myoclonus was reported in tetrahydrobiopterin deficiency, cerebral folate transport deficiency (FOLR1 deficiency), X-linked cobalamin disorder, pyridoxine-dependent and pyridoxal phosphate-responsive epilepsies, methylenetetrahydrofolate reductase deficiency, biotinidase deficiency and Menkes disease. In most of these, myoclonic seizures dominate the presentation.

FOLR1 deficiency arises from biallelic mutations in FOLR1, encoding folate receptor α (FR α), leading to reduced CNS folate levels despite normal systemic concentrations [76]. Affected children present after infancy with developmental delay, speech and language impairment, ataxia, nystagmus, hypotonia, gait abnormalities and myoclonic or tonic seizures. Seizures can be severe, precipitated by fever, or progressing to status epilepticus [76,77]. Behavioral disturbances, including autistic features, may also be present [76]. EEG frequently shows generalized epileptiform discharges or slow spike-wave complexes, consistent with a myoclonic–astatic epilepsy phenotype [78]. MRI reveals white matter hyperintensities and cerebellar atrophy [76,79,80,81]. Diagnosis is confirmed by low CSF 5-methyltetrahydrofolate levels. Treatment with oral or intramuscular 5-formyltetrahydrofolate often improves neurological signs, especially when started early; folic acid is contraindicated, mostly in the case of low but important residual levels of FOLR1 transport activity as it may competitively block FRα [76,77].

Vitamin B6-related epilepsies include pyridoxine-dependent epilepsy (PDE) due to ALDH7A1 deficiency and pyridoxal phosphate-responsive epilepsy (PNPO deficiency) [82,83]. Both typically present neonatally with drug-resistant myoclonic or tonic seizures and burst-suppression EEG patterns. MRI may show non-specific delayed myelination or ventriculomegaly [14,83]. Diagnosis in both conditions rests on demonstrating seizure responsiveness to supraphysiologic pyridoxine or pyridoxal-5′-phosphate (PLP) (PNPO), coupled with biomarkers (e.g., α-AASA in PDE) and confirmatory genetic testing [83]. Lifelong treatment is essential: PDE responds to pyridoxine (typically 30 mg/kg/day up to 500 mg/day) often with excellent seizure control, and adjuncts such as a lysine-restricted diet and arginine supplementation can enhance developmental outcome [84]. PNPO deficiency, by contrast, usually requires lifelong PLP (or pyridoxine) therapy for seizure control in around 60% of cases, though developmental delay remains common if diagnosis is delayed [83]. It is important to remember that certain anti-seizure medications (such as carbamazepine, valproate, phenytoin and phenobarbital) can cause low plasma concentration of PLP and should be avoided [83].

Myoclonus is also well documented in Menkes disease that is caused by mutations in ATP7A and leads to defective copper transport and deficiency of copper-dependent enzymes. Clinically, it may appear as multifocal or generalized myoclonic seizures in early infancy, sometimes alongside focal seizures and later epileptic spasms [85]. Additionally, infants develop neurodegeneration, hypotonia and connective-tissue abnormalities. For diagnosis, beyond copper and ceruloplasmin assays, early testing should include plasma and, where feasible, CSF catecholamine profiling, with elevated dopamine/noradrenaline and DOPAC:DHPG ratios providing sensitive neonatal biomarkers that can be abnormal even before neurological decline [86,87]. MRI may show cerebral and cerebellar atrophy and vascular tortuosity [88]. Treatment with subcutaneous copper-histidine can partially ameliorate symptoms if started early, though efficacy is limited in advanced disease [89,90].

### 3.6. Metabolic Cell Signaling Disorders

This final category includes disorders affecting neurotransmitter synthesis and degradation and endocrine metabolic disorders. Myoclonus occurs most notably in tyrosine hydroxylase deficiency (THD) and succinic semialdehyde dehydrogenase deficiency (SSADH).

THD results from TH mutations disrupting dopamine synthesis [91]. The phenotype includes two major forms, both presenting with hypokinesia, bradykinesia, delayed motor development, oculogyric crises, ptosis and autonomic disturbances [10,91]. Type A usually occurs before the age of 1 and it has a more classical course for DRD: dystonia beginning in lower limbs, progressing to arms, face and the oropharyngeal area. Patients also present with rigidity and tremor. Type B is considered to be more severe, with an onset within the first few weeks of birth, and it develops rapidly and can be manifested by dystonia, tremor and myoclonus. EEG findings are generally non-specific; MRI may show frontotemporal atrophy or ventriculomegaly [92]. Diagnosis relies on CSF neurotransmitter profiling showing low homovanillic acid with normal pterins. Treatment with levodopa combined with other medications such as carbidopa, benzhexol, benserazide, selegiline and biperiden often yields marked improvement, particularly in type A patients [92].

SSADH deficiency, caused by ALDH5A1 mutations, impairs GABA metabolism, leading to accumulation of γ-hydroxybutyric acid [93]. Clinical features include developmental delay, non-progressive encephalopathy, dystonia, dyskinesia hypotonia, ataxia, and seizures—often myoclonic or generalized tonic–clonic. Sleep disturbances, neurobehavioral/psychiatric manifestations such as autism spectrum disorder, attention deficits, obsessive-compulsive behavior and anxiety can also be present [93,94]. MRI demonstrates dentate nucleus, subthalamic nucleus and globus pallidus hyperintensities, and EEG shows diffuse slowing with multifocal spikes [7,94]. Treatment is symptomatic; seizures can be controlled with antiepileptic drugs; however, vigabatrin is not recommended and valproate should be used only in seizures with a drug-resistant generalized spike-wave EEG pattern [94].

## 4. Discussion

This systematic review demonstrates that myoclonus is a recurrent, cross-cutting feature among multiple categories of inherited metabolic diseases. Although the underlying biochemical pathways differ, the pathophysiological mechanisms converge on common neuronal dysfunction—particularly cortical hyperexcitability, impaired inhibitory neurotransmission, and disrupted energy metabolism. The presence of myoclonus, whether isolated or as part of PME, should alert clinicians to possible metabolic etiologies, many of which are amenable to specific treatments. Across the ICIMD classification, myoclonus is especially prevalent in disorders of intermediary and mitochondrial energy metabolism, as well as in lysosomal and vitamin-related diseases. Several disorders, such as phenylketonuria, organic acidemias and CDGs, can be detected by newborn screening, allowing for earlier initiation of treatment and reducing the burden of the disease. The versatile biochemical biomarker for myoclonus is missing due to heterogeneity of its cause. However, choosing a proper diagnostic pathway, according to clinical presentation and family history, may accelerate the diagnosis. Electrophysiologically, cortical myoclonus predominates in mitochondrial and lysosomal disorders, while subcortical and spinal generators are more frequent in lipid and neurotransmitter-related diseases. MRI correlates, including dentate nucleus hyperintensities in CTX and basal ganglia lesions in organic acidurias, further support the concept of region-specific vulnerability linked to metabolic stress. Despite these insights, several limitations persist. Published data remain fragmented, often derived from small series or anecdotal reports. Heterogeneity in diagnostic criteria and lack of unified reporting impede cross-comparison. Furthermore, treatment efficacy is rarely assessed systematically, and long-term outcomes are poorly documented. The establishment of international multicentre registries and standardized neurophysiological protocols would enhance our understanding of prevalence, natural history, and treatment responses. Importantly, the recognition of treatable causes should guide diagnostic algorithms. In every case of unexplained myoclonus, particularly when accompanied by developmental delay, movement abnormalities, or multisystem involvement, clinicians should explore the following metabolic diagnostics: amino acid, lactate and pyruvate analyses from plasma and CSF; analyses of urine organic acids, CSF neurotransmitters and very long-chain fatty acids; lumbar puncture for glucose concentration simultaneously with plasma glucose concentration; acylcarnitine profiling from plasma; analysis of transferrin isoforms; and evaluation of storage disorders. This should be followed by a confirmatory genetic test such as next-generation sequencing.

## 5. Conclusions

Myoclonus constitutes an important and often overlooked manifestation of inherited metabolic diseases. It occurs across virtually all ICIMD categories, reflecting diverse yet converging metabolic mechanisms affecting neuronal excitability. Crucially, several of these conditions are treatable, and early recognition can substantially modify disease trajectory. Clinicians should maintain a high index of suspicion for metabolic causes in any patient presenting with unexplained myoclonus (especially in children and young adults) and promptly initiate biochemical and genetic investigations. Early diagnosis not only enables targeted therapy but also provides families with accurate prognostic and genetic counseling information.

## Data Availability

No new data were generated or analyzed in this study. All data supporting the findings of this review are available within the cited literature.

## Abbreviations

The following abbreviations are used in this manuscript:

5-HIAA	5-Hydroxyindoleacetic Acid
5-HTP	5-Hydroxytryptophan
5-MTHF	5-methyltetrahydrofolate
α-AASA	α-amino adipic semialdehyde
AAV	adenovirus
AHS	Alpers-Huttenlocher Syndrome
Ala	Alanine
ALT	Alanine aminotransferase
ANS	Ataxia neuropathy spectrum
AST	Aspartate aminotransferase
AR-GTPCHD	Autosomal Recessive GTP Cyclohydrolase I Deficiency
BCKA	Branched-Chain Alpha Ketoacids
BCAA	Branched-Chain Amino Acid
CDCA	Chenodeoxycholic acid
CDG	Congenital Disorders of Glycosylation
CLN	Neuronal Ceroid Lipofuscinosis
CNS	Central nervous system
COMT inhibitors	Catechol-O-Methyltransferase Inhibitors
CSF	Cerebrospinal Fluid
CTX	Cerebrotendinous Xanthomatosis
DA	Dopamine
DBS	Dried Blood Spot
DC inhibitor	Dopa Decarboxylase Inhibitor
DD	Developmental Disability
DHPG	Dihydroxyphenylglycol
DHPRD	Dihydropteridine Reductase Deficiency
DOPAC	Dihydroxyphenylacetic acid
DRD	Dopa-responsive Dystonia
ESE	Electrographic Status Epilepticus
FOLR1 deficiency	Cerebral folate transport deficiency
FRα	Folate receptor α
GCH1	Gene encoding GTP Cyclohydrolase I
GGTP	Gamma-glutamyl transferase
GHB	Gamma-Hydroxybutyric Acidura
GI reflux	Gastrointestinal reflux
GLUT1	Glucose transporter type 1
Gly	Glycine
GM1	Gangliosidosis type 1
GM2	Gangliosidosis type 2
GTC	Generalized Tonic–Clonic
HEX	Hexoaminidase
HPA	Hyperphenylalaninemia
HVA	Homovanillic Acid
ICIMD	International Classification of Inherited Metabolic Disorders
ID	Intellectual Disability
IEMs	Inherited errors of metabolism
JME	Juvenile myoclonic epilepsy
MAO	Monoamine Oxidase
MEMSA	Myoclonic epilepsy myopathy sensory ataxia
MPI	Mannose Phosphate Isomerase
NGLY1	N-glycanase type 1
NPC	Niemman-Pick disease type C
P1	Patients with GTP Cyclohydrolase I Deficiency
PCBD1	gene encoding Pterin-4a-Carbinolamine Dehydratase
PCDD	Pterin-4a-Carbinolamine Dehydratase Deficiency
PDE	Pyridoxine-dependent epilepsy
PED	paroxysmal exercise-induced dyskinesia
Phe	Phenylalanine
PLGA	NPs Poly(lactic-co-glycolic acid) nanoparticles
PLP	Pyridoxal 5′-phosphate
PME	Progressive myoclonic epilepsy
POLG	Polymerase gamma
PNPO	Pyridox(am)ine 5′-phosphate oxidase
PTPSD	6-Pyruvoyl-Tetrahydropterin Synthase Deficiency
PTS	gene encoding 6-Pyruvoyl-Tetrahydropterin Synthase
QDPR g	gene encoding Quinoid Dihydropteridine Reductase
SCAE	Spino-cerebellar ataxia with epilepsy
SEP	Somatosensory Evoked Potential
Ser	Serine
SLEs	Stroke-Like Episodes
SPR	gene encoding Sepiapterin Reductase
SR	Sepiapterin Reductase Deficiency
SSADH	Succinic Semialdehyde Dehydrogenase Deficiency
SSRIs	Selective Serotonin Reuptake Inhibitors
THD	Tyrosine Hydroxylase Deficiency
IUGR	Intrauterine Growth Retardation
VA	Valproic Acid
